# The Keap1–Nrf2 system in cancers: stress response and anabolic metabolism

**DOI:** 10.3389/fonc.2012.00200

**Published:** 2012-12-26

**Authors:** Yoichiro Mitsuishi, Hozumi Motohashi, Masayuki Yamamoto

**Affiliations:** ^1^Department of Medical Biochemistry, Tohoku University Graduate School of MedicineSendai, Japan; ^2^Department of Respiratory Medicine, Tohoku University Graduate School of MedicineSendai, Japan; ^3^Center for Radioisotope Sciences, Tohoku University Graduate School of MedicineSendai, Japan; ^4^Center for Regulatory Epigenome and Diseases, Tohoku University Graduate School of MedicineSendai, Japan

**Keywords:** stress response, redox homeostasis, transcription, purine nucleotide, glutathione

## Abstract

The Keap1–Nrf2 [Kelch-like ECH-associated protein 1–nuclear factor (erythroid-derived 2)-like 2] pathway plays a central role in the protection of cells against oxidative and xenobiotic stresses. Nrf2 is a potent transcription activator that recognizes a unique DNA sequence known as the antioxidant response element (ARE). Under normal conditions, Nrf2 binds to Keap1 in the cytoplasm, resulting in proteasomal degradation. Following exposure to electrophiles or reactive oxygen species, Nrf2 becomes stabilized, translocates into the nucleus, and activates the transcription of various cytoprotective genes. Increasing attention has been paid to the role of Nrf2 in cancer cells because the constitutive stabilization of Nrf2 has been observed in many human cancers with poor prognosis. Recent studies have shown that the antioxidant and detoxification activities of Nrf2 confer chemo- and radio-resistance to cancer cells. In this review, we provide an overview of the Keap1–Nrf2 system and discuss its role under physiological and pathological conditions, including cancers. We also introduce the results of our recent study describing Nrf2 function in the metabolism of cancer cells. Nrf2 likely confers a growth advantage to cancer cells through enhancing cytoprotection and anabolism. Finally, we discuss the possible impact of Nrf2 inhibitors on cancer therapy.

## INTRODUCTION

In our daily lives, we are constantly exposed to miscellaneous chemical and physical insults, including environmental pollutants, food additives, naturally occurring plant alkaloids, ultraviolet and ionizing radiation. In addition to these external stresses, there are many intrinsic toxicants produced during physiological metabolism and pathological processes, including reactive oxygen species (ROS) and proinflammatory cytokines. All aerobic organisms are fundamentally dependent on oxygen, which enables efficient energy production and provokes the oxidative damage of cellular components. To contend with these insults, our bodies are equipped with a cytoprotective mechanism for survival.

**FIGURE 1:**
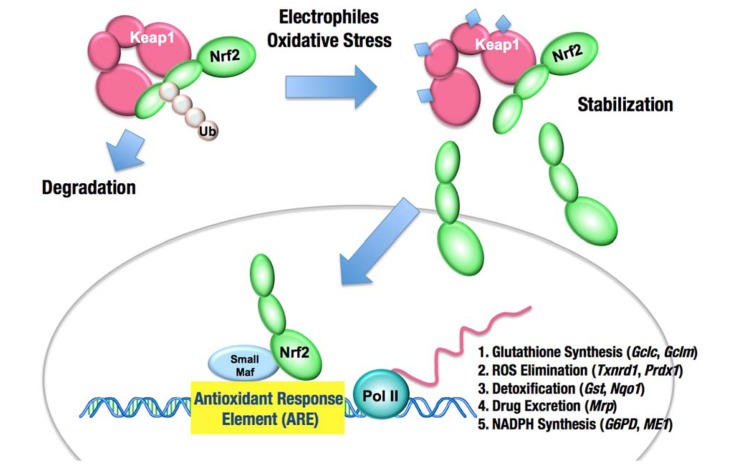
**The Keap1–Nrf2 system**. Under normal conditions, Nrf2 is constantly ubiquitinated through Keap1 and degraded in the proteasome. Following exposure to electrophiles or oxidative stress, Keap1 is inactivated. Stabilized Nrf2 accumulates in the nucleus and activates many cytoprotective genes. Ub, ubiquitin.

The Keap1–Nrf2 [Kelch-like ECH-associated protein 1–nuclear factor (erythroid-derived 2)-like 2] system is one of the most critical cytoprotective mechanisms acquired in vertebrates over the course of evolution (Morita and Motohashi, 2011). The transcription factor Nrf2 is a potent transcriptional activator that plays a central role in the inducible expression of many cytoprotective genes in response to oxidative and electrophilic stresses (Itoh et al., 1997; Ishii et al., 2000). Belonging to the Cap “n” collar (CNC) family of transcription factors, Nrf2 possesses a well-conserved basic region-leucine zipper (bZip) motif and binds to the antioxidant response element (ARE), or electrophile response element (EpRE; TGA(G/C)NNNGC), through heteromerizing with the small Maf protein (**Figure 1**).

Keap1 is essential for the regulation of Nrf2 activity (Itoh et al., 1999). Under normal conditions, Nrf2 is constantly ubiquitinated through Keap1 in the cytoplasm and degraded in the proteasome. Upon exposure to electrophiles or ROS, Keap1 is inactivated and Nrf2 is stabilized. Consequently, Nrf2 translocates into the nucleus and activates the transcription of many cytoprotective genes that encode detoxifying enzymes and antioxidant proteins, including NAD(P)H:quinone oxidoreductase 1, glutathione *S*-transferase, and heme oxygenase-1. In general, the target genes of Nrf2 are involved in glutathione synthesis, the elimination of ROS, xenobiotic metabolism, and drug transport. Nrf2 coordinately activates these genes and exerts a protective function against xenobiotic and oxidative stresses (**Figure 1;** Moinova and Mulcahy, 1999; McMahon et al., 2001; Chanas et al., 2002; Solis et al., 2002; Thimmulappa et al., 2002; Bea et al., 2003; Sekhar et al., 2003; MacLeod et al., 2009). Recent studies have shown that Nrf2 is a potent cell survival factor and enhances proliferation of cancers.

## NRF2 IS A CYTOPROTECTIVE FACTOR AND BENEFICIAL FOR THE HEALTH

Important roles for Nrf2 in the protection from xenobiotic and oxidative stresses have been shown in the analyses of *Nrf2*-null mice. *Nrf2*-null mice suffer from acute hepatotoxicity after acetaminophen exposure (Enomoto et al., 2001). The exposure to diesel exhausts increases the formation of DNA adducts in *Nrf2*-null mice (Aoki et al., 2001). *Nrf2*-null mice are more susceptible to cigarette smoke-induced emphysema (Rangasamy et al., 2004), bleomycin-induced pulmonary fibrosis (Cho et al., 2004), and hyperoxic lung injury (Cho et al., 2002b). A single-nucleotide polymorphism (SNP) in the promoter region of the mouse *Nrf2* gene has been linked to the reduced expression of *Nrf2* and subsequent lung damage caused by hyperoxia (Cho et al., 2002a). The human *NRF2* gene also harbors SNPs in the promoter region (Yamamoto et al., 2004), which have been linked to the risk of acute lung injury (Marzec et al., 2007). These data demonstrate that Nrf2 significantly contributes to the protection against extrinsic insults.

Nrf2 also plays an important role in the response to intrinsic oxidative stress. Cellular capacities for ROS elimination are limited in *Nrf2*-null mice (Hirayama et al., 2003). Accordingly, *Nrf2*-null mice tend to spontaneously develop various inflammatory disorders, including glomerulonephritis, immune-mediated hemolytic anemia, and multi-organ autoimmune inflammation (Yoh et al., 2001; Lee et al., 2004; Ma et al., 2006). The chronic accumulation of intracellular ROS seems to underlie the pathogenesis of these disorders. Thus, Nrf2 also critically contributes to the protection from intrinsic insults.

One of the most important characteristics of Nrf2-mediated transcription is the inducibility in response to xenobiotic and oxidative stresses. Under normal conditions, the activity of the Nrf2-mediated transcription is low, as most of Nrf2 protein is degraded in the proteasome (Itoh et al., 2003). When cells are exposed to electrophiles or ROS, Nrf2 is stabilized and accumulates in the nucleus, which results in the robust activation of Nrf2 target genes. Thus, the mechanism underlying Nrf2 degradation under normal conditions and the stabilization of Nrf2 following exposure to stress are critical clues to the revelation of the molecular basis of our defense system.

## REGULATORY MECHANISMS OF NRF2 ACTIVITY

**FIGURE 2:**
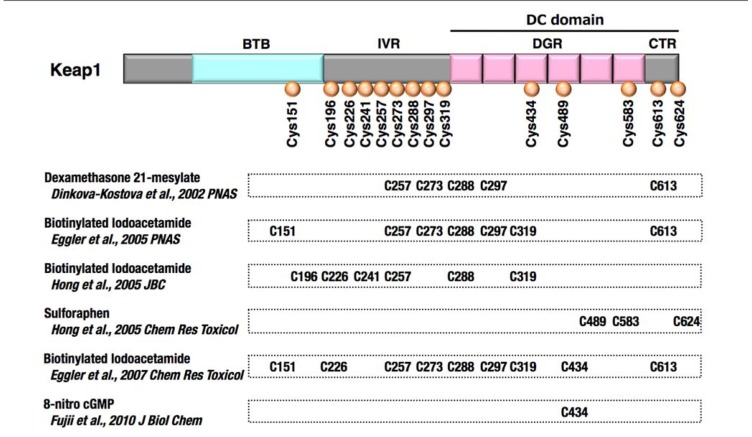
**Keap1 is a thiol-rich protein that is sensitive to electrophilic covalent modification**. Domain structure of Keap1 is shown, and reactive cysteine residues are indicated with brown circles. Direct modification of cysteine residues was demonstrated using various electrophiles. Each electrophile attacks a unique set of cysteines.

Keap1 was identified as a cytoplasmic Nrf2-interacting protein that negatively regulates Nrf2 activity (Itoh et al., 1999). In the absence of Keap1, Nrf2 is constitutively stabilized, and the expression of Nrf2 target genes is maintained at high levels (Wakabayashi et al., 2003). Possessing a BTB domain at the N-terminal region, Keap1 serves as an adaptor for the Cullin 3-based ubiquitin E3 ligase for Nrf2 (Cullinan et al., 2004; Kobayashi et al., 2004; Zhang et al., 2004; Furukawa and Xiong, 2005). Keap1 is a thiol-rich protein that possesses multiple highly reactive cysteine residues (**Figure 2**). Electrophiles directly modify the cysteine residues, leading to Keap1 inactivation, Nrf2 stabilization, and the induction of many cytoprotective genes (Dinkova-Kostova et al., 2002; Levonen et al., 2004; Sakurai et al., 2006; Rachakonda et al., 2008; Fujii et al., 2010). High concentrations of electrophiles or highly potent electrophiles covalently modify not only Keap1 but also various cellular components, such as nucleic acids, proteins, and lipids, which adversely affects the cellular function. Because of the ultra-sensitive nature of Keap1 cysteine residues, the Keap1–Nrf2 system responds to low levels of electrophiles or less potent electrophiles and induces the cytoprotective machineries for the prevention of cellular damages.

**FIGURE 3:**
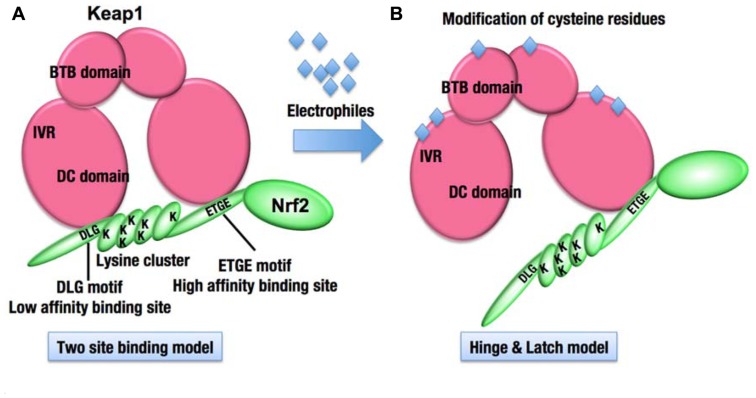
**Molecular mechanism of stress sensing in the Keap1–Nrf2 system**. **(A)** The Keap1 homodimer binds one molecule of Nrf2. The ETGE and DLG motifs of Nrf2 represent high and low affinity binding sites, respectively. The lysine residues (K) are clustered between the two motifs and represent ubiquitination targets. **(B)** The modification of cysteine residues in Keap1 with electrophiles is expected to modify the overall conformation of the Keap1 homodimer, resulting in the termination of Nrf2 ubiquitination.

**FIGURE 4:**
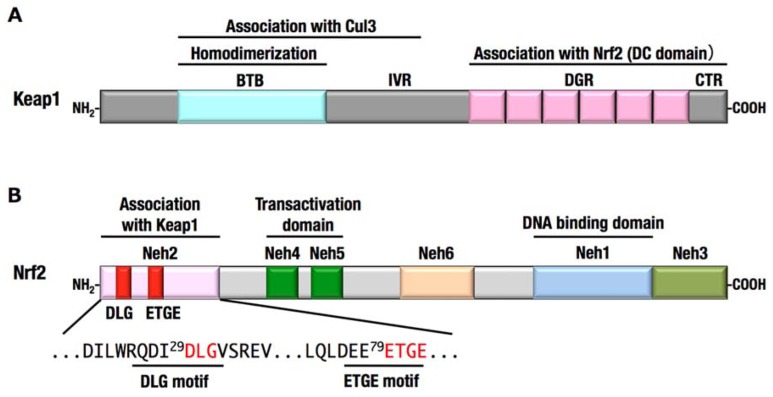
**Domain structures of Keap1 **(A)** and Nrf2 **(B)**.**(A)**** The N-terminal region of Keap1 mediates homodimerization and association with Cul3, and the C-terminal region of Keap1 mediates binding with Nrf2. **(B)** The N-terminal region of Nrf2 is designated Neh2 domain, which contains two motifs, DLG and ETGE, responsible for the interaction with Keap1. Neh4, Neh5, and Neh3 domains are important for the transactivation activity of Nrf2 (Katoh et al., 2001; Nioi et al., 2005). Neh6 domain contains the phosphodegron that is recognized by β-TrCP. Neh1 domain is a basic-region leucine zipper motif for DNA binding and dimerization with small Maf.

The results of biochemical and structural analyses revealed the overall structure of Keap1–Nrf2 complex under the normal condition. Two molecules of Keap1 form a homodimer through the N-terminal BTB domain, and the C-terminal globular domains, called the DC domains, are positioned apart from each other (Ogura et al., 2010; **Figures 3A and 4A**). Two DC domains of the Keap1 homodimer associate with one molecule of Nrf2 (Tong et al., 2006). The N-terminal region of Nrf2, called the Neh2 domain, bridges the two DC domains at two separate binding sites, namely the ETGE and DLG motifs (**Figure 4B**). The lysine residues serve as ubiquitination target sites and are clustered in the Neh2 domain between the ETGE and DLG motifs. The two-site binding between Keap1 and Nrf2 appears to be favorable for the efficient ubiquitination of Nrf2.

Interestingly, the affinity between the ETGE motif and the Keap1 DC domain is much higher than that between the DLG motif and the Keap1 DC domain (Tong et al., 2006). The Keap1 cysteine residue-mutant and electrophile-modified Keap1, both of which have lost the ability to ubiquitinate Nrf2, often retain the capacity to bind with Nrf2. A unique model emerging from these observations is the “hinge and latch” model (Tong et al., 2007; **Figure 3B**). The cysteine modification is expected to trigger a conformational change of the Keap1 homodimer, which could dissociate the weak binding site, the DLG motif (latch), from the Keap1 DC domain, while the strong binding site, the ETGE motif (hinge), remains attached to the other Keap1 DC domain. The hinge and latch model is one of the attractive hypotheses for the sensing mechanism of the Keap1–Nrf2 system.

Besides the cysteine modification of Keap1, phosphorylation of Nrf2 regulates the activation of Nrf2. Protein kinase C phosphorylates Ser40 of Nrf2, resulting in the activation of Nrf2 (Huang et al., 2002). Nrf2 is also activated by PERK-dependent phosphorylation and promotes cell survival under endoplasmic reticulum stress (Cullinan et al., 2003). In contrast, Nrf2 phosphorylation by GSK-3β at serine residues of Neh6 domain (**Figure 4B**) inhibits its activity by promoting β-TrCP-mediated ubiquitination and degradation of Nrf2 (Rada et al., 2012). This is a second degradation pathway of Nrf2, which seems to be independent of Keap1.

## NRF2 INDUCERS ARE EFFECTIVE FOR CANCER CHEMOPREVENTION

Because Nrf2 activates many genes encoding detoxification enzymes, Nrf2 deficiency exacerbates the formation of DNA adducts, which increases the risk of carcinogenesis. Conversely, the increased activity of Nrf2 is effective for the prevention of chemical carcinogenesis. Oltipraz (4-methyl-5-[2-pyrazinyl]-1,2-dithiole-3-thione) is an Nrf2 inducer that suppresses the benzo[a]pyrene-induced gastric cancer formation, which is not observed in *Nrf2*-null mice (Ramos-Gomez et al., 2001). Thus, the antitumor effect of oltipraz requires Nrf2 function. *N*-nitrosobutyl (4-hydroxybutyl) amine (BBN) causes urinary bladder carcinogenesis, and this effect was also suppressed through oltipraz in an Nrf2-dependent manner (Iida et al., 2004). The results of a field study in China showed that the Nrf2 inducer, sulforaphane, which is contained in broccoli sprouts, is potentially effective for cancer chemoprevention (Wang et al., 1999; Kensler et al., 2005).

Recent studies revealed that Wilms tumor gene on the X chromosome (WTX) and PALB2, a major BRCA2 binding partner known as FANCN, have been shown to interact with the DC domain of KEAP1, inhibit the ubiquitination of Nrf2 and promote NRF2-dependent transcription (Camp et al., 2012; Ma et al., 2012). Functional defects of these gene products enhance the ubiquitination of Nrf2, resulting in the decreased activity of Nrf2. *WTX* and *PALB2* are both considered as tumor suppressor genes since their mutations are often found in kidney tumor (Wilms tumor) and breast and pancreatic cancers, respectively. WTX and PALB2 may suppress carcinogenesis partly through maintaining the Nrf2 activity for cytoprotection.

## CANCER CELLS OFTEN HIJACK THE KEAP1–NRF2 SYSTEM

Intriguingly, various human cancers frequently exhibit increased levels of NRF2 (Singh et al., 2006; Shibata et al., 2008a,b; Wang et al., 2008a; Kim et al., 2010; Solis et al., 2010; Zhang et al., 2010; Taguchi et al., 2011). Highly activated NRF2 target genes, encoding detoxification and antioxidant enzymes, confer a great advantage to cancer cells for survival against anti-cancer drugs and irradiation (Wang et al., 2008b; Singh et al., 2010; Zhang et al., 2010). Constitutively stabilized NRF2 also promotes cell proliferation, as *NRF2* knockdown inhibits the proliferation of human lung cancer cell lines (Singh et al., 2008). Cancer cells hijack the KEAP1–NRF2 system, acquiring malignant properties. Indeed, the prognoses of patients carrying NRF2-positve cancers are significantly poor (Shibata et al., 2008b; Solis et al., 2010; Inoue et al., 2012).

**FIGURE 5:**
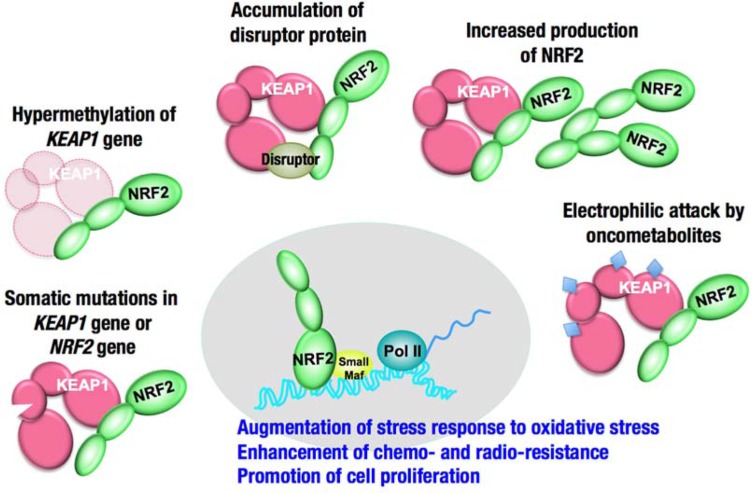
**Increased activity of NRF2 in cancer cells**. The degradation of NRF2 is inhibited in some cases, and the production of NRF2 is increased in other cases.

Several mechanisms have been reported for the increased activity of NRF2 in cancers (**Figure 5**): (1) somatic mutations in *KEAP1* or *NRF2*, (2) DNA hypermethylation at the promoter region of *KEAP1*, (3) the aberrant accumulation of proteins that disrupt the KEAP1–NRF2 interaction, (4) transcriptional up-regulation of *NRF2* gene through oncogene-dependent signaling, and (5) the modification of KEAP1 protein through oncometabolites. A detailed description of each mechanism is provided below.

### SOMATIC MUTATIONS IN *KEAP1* OR *NRF2*

**FIGURE 6:**
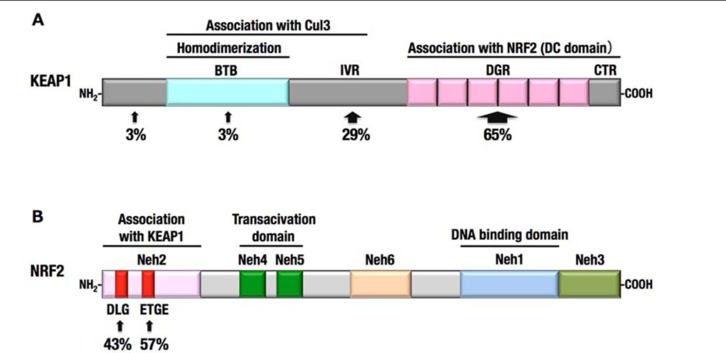
**Somatic mutations in *KEAP1* and *NRF2* genes identified in human cancers**. **(A)** More than half of the *KEAP1* gene mutations were identified in the DC domain. **(B)** All *NRF2* gene mutations were restricted to the DLG and ETGE motifs.

**FIGURE 7:**
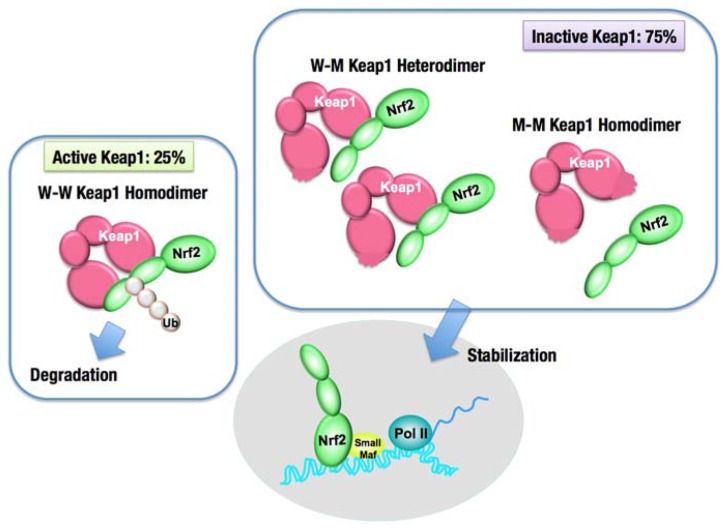
**Dominant negative effect of the *Keap1* gene mutation**. The intact Keap1 homodimer ubiquitinates Nrf2, while the Keap1 dimer, containing one or two mutant Keap1 proteins, cannot ubiquitinate Nrf2. A single allele mutation in the *Keap1* gene results in the production of an equal molar ratio of the wild-type Keap1 (W) to the mutant Keap1 (M). Keap1 dimerization generates three kinds of dimers, W–W, W–M, and M–M, by 1:2:1 ratio. Since W–M and M–M dimers are incapable of ubiquitinating Nrf2, the overall Keap1 activity is reduced by 75%, and consequently, Nrf2 is stabilized.

Missense mutations in the *KEAP1* gene have been identified in several human cancers, particularly in solid tumors in the lung, gallbladder and liver (Padmanabhan et al., 2006; Singh et al., 2006; Nioi and Nguyen, 2007; Ohta et al., 2008; Shibata et al., 2008a; Takahashi et al., 2010; Li et al., 2011). Somatic mutations cause amino acid substitutions; thus, the resultant KEAP1 mutant proteins are not able to fulfill the adaptor function of the E3 ubiquitin ligase for NRF2. More than half of the *KEAP1* mutations that have been reported so far are distributed in the DC domain, which is essential for association with NRF2 (Taguchi et al., 2011; **Figure 6A**). Interestingly, heterozygous *KEAP1* mutations frequently occur in lung cancers (Padmanabhan et al., 2006; Singh et al., 2006; Ohta et al., 2008; Shibata et al., 2008a). An elegant mouse model demonstrated that a heterozygous mutation in the *KEAP1* gene is sufficient to reduce KEAP1 activity and consequently stabilize NRF2 (Suzuki et al., 2011; **Figure 7**). Based on the observation that Keap1 functions as a homodimer, the heterozygous missense mutation generates three types of Keap1 dimers, i.e., wild-type homodimer, wild-type-mutant heterodimer, and mutant homodimer at a ratio of 1:2:1. Because the hinge and latch hypothesis predicts that the wild-type-mutant heterodimer does not support Nrf2 ubiquitination, a heterozygous missense mutation would result in the 75% reduction of Keap1 activity. The results of a study concerning the graded expression of the Keap1 gene in mice demonstrated that a 50% reduction of Keap1 activity does not induce Nrf2 accumulation, whereas a 75% reduction is enough to elicit this effect (Taguchi et al., 2010). Thus, the heterozygous *KEAP1* mutation conferring the growth advantage on cancers is consistent with the two-site binding model and hinge and latch model of the Keap1–Nrf2 system.

Mutations in *NRF2* gene were also identified in several cancers, including lung, head and neck, and esophageal cancers (Shibata et al., 2008b, 2011; Kim et al., 2010). Notably, all the mutations in the *NRF2* gene are clustered within the DLG (43%) and ETGE (57%) motifs (**Figure 6B**), which are critical sites for the binding of Nrf2 to the Keap1 DC domain. Mutations in the ETGE motif disrupt the high-affinity binding of Keap1 with Nrf2; thus, ETGE mutant proteins are not ubiquitinated and accumulate in the nucleus (Shibata et al., 2008b). Mutations in the DLG motif disrupt the low-affinity binding of Keap1 with Nrf2, resulting in the stabilization of Nrf2, although the association between Keap1 and Nrf2 through the high-affinity binding site, ETGE, is retained.

### DNA HYPERMETHYLATION AT THE PROMOTER REGION OF *KEAP1* GENE

Epigenetic alterations also facilitate NRF2 stabilization. The hypermethylation of the promoter region of *KEAP1* gene has been identified in cancer cells generated in lung (Wang et al., 2008a; Muscarella et al., 2011b), prostate (Zhang et al., 2010), malignant glioma (Muscarella et al., 2011a), and colorectal cancers (Hanada et al., 2012). The inhibition of *KEAP1* gene expression results in NRF2 accumulation, conferring a survival and growth advantage to cancer cells. The epigenetic abnormalities in the *KEAP1* gene in lung cancers and malignant gliomas are indeed associated with poor clinical outcomes (Muscarella et al., 2011a,b).

### ABERRANT ACCUMULATION OF PROTEINS THAT DISRUPT THE KEAP1–NRF2 INTERACTION

Several non-electrophilic inducers of Nrf2 originating from endogenous stress have been identified. For instance, the cyclin-dependent kinase inhibitor p21, which is a p53-regulated gene product with pro-survival properties, has been shown to associate with the DLG motif of Nrf2 (Chen et al., 2009). Consequently, the two-site binding between Keap1 and Nrf2 is disrupted, and the E3 ligase activity of the Keap1-Cul3 complex is inactivated. Indeed, the activation of cytoprotective genes through Nrf2 is more augmented in the presence than in the absence of p21. As a downstream effector of p53 that mediates cell cycle arrest and apoptosis (Gartel and Tyner, 2002), p21 also promotes cell survival in response to oxidative stress (O’Reilly, 2005). Thus, the cytoprotective function of p21 may be dependent on Nrf2.

Another example of a disruptor protein is SQSTSM1/p62, which is a polyubiquitin binding protein and targets various substrates for autophagy (Komatsu and Ichimura, 2010). The STGE motif within p62 binds to Keap1 DC pockets with a similar affinity as that of the DLG motif of Nrf2 (Komatsu et al., 2010; Lau et al., 2010). When autophagy is impaired, increased p62 competes with the DLG motif for binding to the Keap1 DC pocket and inhibits the ubiquitination of Nrf2, causing Nrf2 stabilization and the increased expression of cytoprotective genes. Importantly, the abnormal accumulation of p62 is often observed in certain cancers, such as hepatocellular carcinoma (Lu et al., 2001; Strnad et al., 2008; Inami et al., 2011), which suggests that the increased activity of NRF2 might contribute to the malignant progression of these cancers.

### TRANSCRIPTIONAL UP-REGULATION OF *NRF2* GENE BY ONCOGENE-DEPENDENT SIGNALING

Because the degradation process primarily regulates the level of Nrf2 protein, there have been a few studies concerning the transcriptional control of the *Nrf2* gene. The results of a recent insightful study implicate the transcriptional control of the *Nrf2* gene as a determinant of Nrf2 activity (DeNicola et al., 2011). The oncogene *K-Ras* activates *Nrf2* transcription through the Mek-Erk-Jun signaling pathway and reduces the ROS levels in primary fibroblasts. The transcriptional activation of *Nrf2* is also suggested as a part of the tumorigenic activity of other oncogene products, such as Braf and c-Myc. Thus, oncogene activation is likely to increase the expression of NRF2, negating the need for mutations in *NRF2* or *KEAP1*. A larger spectrum of cancers might utilize this non-mutational pathway to create a reducing environment that enables tumor promotion.

### MODIFICATION OF KEAP1 PROTEIN THROUGH ONCOMETABOLITES

While unique metabolic activities in cancers, such as aerobic glycolysis, have long been recognized (Koppenol et al., 2011), recent technical advances have accelerated the progress in the field of cancer metabolism. The identification of 2-hydroxyglutarate as an aberrant metabolite produced by mutant IDH1 or IDH2 enzymes in glioma and acute myeloid leukemia established a new concept of oncometabolites (Dang et al., 2009; Ward et al., 2010). Oncometabolites are unique metabolites in cancer cells that are involved in the initiation and/or progression of cancers.

Fumarate, one of the intermediates of the TCA cycle, is considered to be an oncometabolite that promotes cancer based on the human cases carrying heterozygous germline mutations in the *fumarate hydratase* (*FH*) gene. These patients exhibit elevated levels of fumarate and develop hereditary leiomyomatosis and renal cell cancer (HLRCC), a syndrome characterized by smooth muscle tumors and papillary renal cell carcinoma type 2 (pRCC-2; Tomlinson et al., 2002). Interestingly, the Nrf2-mediated antioxidant response pathway is highly activated in FH-mutant cells (Adam et al., 2011; Ooi et al., 2011). It was revealed that fumarate, possessing a weak electrophilic property, modifies KEAP1 cysteine residues, resulting in the stabilization of NRF2. Heme oxygenase 1 (HO-1) is an NRF2 target gene that prevents FH deficiency-mediated succinate stagnation through the heme synthesis and degradation pathway (Frezza et al., 2011). The inhibition of HO-1 is effective for the suppression of FH-deficient cancers. The contribution of other NRF2 target gene products to the properties of FH-deficient cancers would be studied in more detail.

## BEYOND REGULATING REDOX HOMEOSTASIS: NRF2 PROMOTES ANABOLIC PATHWAYS IN CANCERS

**FIGURE 8:**
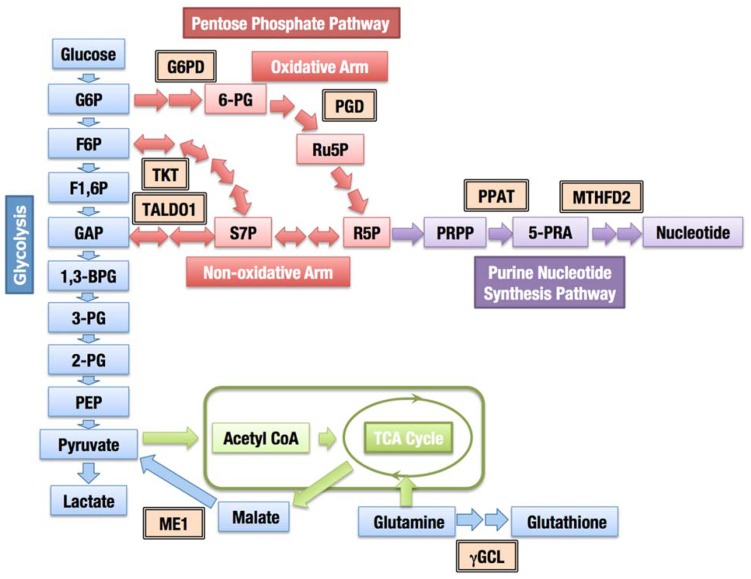
**Contribution of Nrf2 to cellular metabolism**. The enzymes regulated through Nrf2 are indicated with double-framed boxes (G6PD, glucose-6-phosphate dehydrogenase; PGD, phosphogluconate dehydrogenase; TKT, transketolase; TALDO1, transaldolase 1; PPAT, phosphoribosyl pyrophosphate amidotransferase; MTHFD2, methylenetetrahydrofolate dehydrogenase 2; ME1, malic enzyme 1; γGCL, γ-glutamylcysteinyl ligase). Abbreviations of metabolites; 1,3-BPG, 1,3-bisphosphoglycerate; 2-PG, 2-phosphoglycerate; 3-PG, 3-phosphoglycerate; 6-PG, 6-phosphogluconate; 5-PRA, β-5-phosphorybosylamine; F1,6P, fructose 1,6-bis-phosphate; F6P, fructose 6-phosphate; G6P, glucose 6-phosphate; GAP, glyceraldehyde 3-phosphate; PEP, phosphoenolpyruvate; PRPP, phosphoribosyl phosphate; R5P, ribose 5-phosphate; Ru5P, ribulose 5-phosphate; S7P, sedoheptulose 7-phosphate.

**FIGURE 9:**
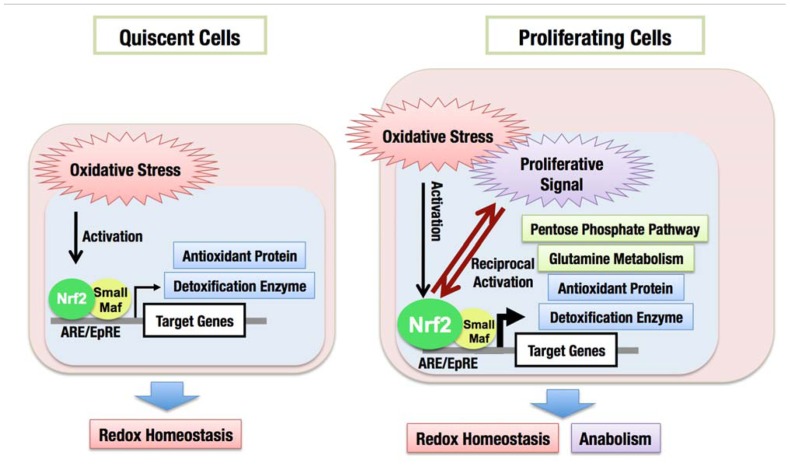
**Functional expansion of Nrf2 in proliferating cells**. In quiescent cells, Nrf2 is activated in response to oxidative stress and induces the expression of cytoprotective genes encoding antioxidant proteins and detoxification enzymes, which maintains the cellular redox homeostasis. In proliferating cells, Nrf2 activity is augmented especially under the sustained activation of PI3K–Akt pathway. Nrf2 activates metabolic genes in addition to cytoprotective genes, resulting in the redirection of glucose and glutamine into anabolic pathway, which is advantageous for cell proliferation.

In addition to coordinately activating the genes encoding detoxifying enzymes and antioxidant proteins, the constitutive accumulation of Nrf2 confers chemo- and radio-resistance for cancer cell survival. Then, what is the role of Nrf2 in promoting cell proliferation in the absence of external insults? We recently found that Nrf2 redirects glucose and glutamine into anabolic pathways and promotes metabolic activities that are advantageous for proliferation (Mitsuishi et al., 2012). An attempt to identify Nrf2 target genes in cancer cells revealed that Nrf2 directly activates the genes whose products are involved in the pentose phosphate pathway and nicotinamide adenine dinucleotide phosphate (NADPH) production, such as glucose-6-phosphate dehydrogenase (G6PD), phosphogluconate dehydrogenase (PGD), transketolase (TKT) and transaldolase 1 (TALDO1), and malic enzyme 1 (ME1; **Figure 8**). The metabolite analysis demonstrated that Nrf2 strongly promotes purine nucleotide synthesis, resulting in the increased production of purine nucleotides. Nrf2 also promotes glutamine consumption through enhancing glutaminolysis and glutathione synthesis (Figure 8). Importantly, the effects of Nrf2 on gene expression and metabolic activities are obvious under the sustained activation of PI3K–Akt signaling pathway. The functional expansion of Nrf2 in proliferating cells directs the enhancement of anabolic metabolism, maintains redox homeostasis and further promotes the activation of PI3K–Akt signaling, suggesting the presence of positive feedback between Nrf2 and the PI3K–Akt pathway in proliferating cells (Figure 9).

In good agreement with this observation, a simple accumulation of Nrf2 is not sufficient for the development of spontaneous cancers (Taguchi et al., 2010), although a large number of human cancers depend on Nrf2 activity. In *Keap1* knockdown (*Keap1*^KD^) mice, the Keap1 mRNA level is reduced to approximately 5% of that in wild-type mice, and constitutive Nrf2 activation is observed in various tissues, such as liver, lung, and kidney. However, *Keap1*^KD^ mice did not develop any spontaneous cancers. Thus, increased Nrf2 activity does not initiate cancer development but confers advantages in terms of proliferation and stress resistance once a cell acquires uncontrolled proliferative properties. Nrf2 is a critical survival factor for cancer cells, which is best described as a form of non-oncogene addiction.

The pentose phosphate pathway fulfills two important cellular requirements. The first requirement is to generate ribose 5-phosphate for the synthesis of nucleotides, and the other is to provide reducing power in the form of NADPH. Ribose 5-phosphate is a nucleotide precursor, which is indispensable for proliferating cells and generated through two distinct pathways, the oxidative and non-oxidative arms of the pentose phosphate pathway (Figure 8). The oxidative arm is an irreversible mechanism associated with the production of NADPH. The activity of G6PD, one of the enzymes of the oxidative pathway, was associated with thymidine incorporation, indicating a critical role for G6PD in cell growth (Tian et al., 1998). The balance between the need for NADPH or ribose 5-phosphate determines the direction of the non-oxidative arm (Wamelink et al., 2008). When the requirement for NADPH production dominates, pentose phosphates produced from the oxidative arm are recycled back to glycolytic intermediates. When a large quantity of nucleotides is required, such as in cancer cells, both the oxidative and non-oxidative arms are directed toward ribose 5-phosphate production (Boros et al., 2000). The increased expression of one of the enzymes involved in the non-oxidative pathway, TKTL1, was associated with the poor prognosis of colon and urothelial cancers (Langbein et al., 2006), suggesting that the non-oxidative arm is also critical for the malignant growth of some cancers. The inhibition of the TKTL1 activity has been shown to repress the proliferation of hepatoma cells (Zhang et al., 2007).

Nrf2 not only increases the enzyme levels of both the oxidative and non-oxidative arms, but it also facilitates the utilization of ribose 5-phosphate for the purine nucleotide synthesis (Mitsuishi et al., 2012), which appears to maintain the ribose 5-phosphate concentration at a low level and efficiently divert glucose flux into purine nucleotide synthesis through both arms of the pentose phosphate pathway. Although Nrf2 does not directly contribute to aerobic glycolysis, glucose uptake and glycolytic activity are stimulated under the sustained activation of the PI3K–Akt signaling (Elstrom et al., 2004; Wieman et al., 2007), thereby increasing the supply of glycolytic intermediates. Thus, Nrf2 accumulation and activation of PI3K–Akt pathway achieve the efficient synthesis of the purine nucleotides.

The oncoprotein c-Myc regulates nucleotide metabolism (Mannava et al., 2008). c-Myc directly activates the genes involved in nucleotide synthesis, including thymidylate synthase for pyrimidine metabolism, inosine monophosphate dehydrogenase 1 and 2 for purine metabolism, and phosphoribosyl pyrophosphate (PRPP) synthetase 2 for the production of PRPP, which is a common precursor for purine and pyrimidine nucleotides. While purine nucleotide synthesis is selectively affected through Nrf2 activation, c-Myc is involved in the regulation of both purine and pyrimidine nucleotide synthesis.

## OTHER REGULATORS OF THE PENTOSE PHOSPHATE PATHWAY

It has been shown that the activation of mammalian target of rapamycin complex 1 (mTORC1) increases the metabolic flux through both glycolysis and the oxidative arm of the pentose phosphate pathway (Düvel et al., 2010). Sterol regulatory element-binding proteins (SREBP1 and SREBP2) have been suggested as one of the downstream effectors of mTORC1, which are responsible for the regulation of the pentose phosphate pathway enzymes. Nrf2 efficiently activates the pentose phosphate pathway genes in the active PI3K–Akt pathway, where mTORC1 and SREBP are also activated; therefore, Nrf2 and SREBP might synergistically facilitate the oxidative arm of the pentose phosphate pathway.

In contrast, the non-oxidative arm of the pentose phosphate pathway is enhanced in the presence of oncogenic Kras (Kras^G12D^) in pancreatic tumors (Ying et al., 2012). Myc was suggested as a downstream effector of Kras^G12D^ in this study. Because the Kras^G12D^-Myc axis has been suggested to induce Nrf2 gene expression and increased the activity of Nrf2 in pancreatic cancers (DeNicola et al., 2011), the activation of the non-oxidative arm of the pentose phosphate pathway might depend on Nrf2.

Another unique regulator of the pentose phosphate pathway is the tumor suppressor p53, which inhibits the pentose phosphate pathway through binding to G6PD and preventing the formation of the active dimer (Jiang et al., 2011). Consequently, wild-type p53 suppresses NADPH production, whereas tumor-associated p53 mutants show almost no activity in inhibiting G6PD, thereby maintaining a high level of NADPH production. The Nrf2-mediated induction of the pentose phosphate pathway at the transcription level would substantially increase the pathway activity in p53-mutated cancers.

### DETOXIFICATION OF ROS FOR CELL SURVIVAL AND PROLIFERATION

The glutathione synthesis pathway is another important anabolic target of Nrf2 (MacLeod et al., 2009). Glutathione is a key molecule for redox homeostasis, and the reduced form of glutathione is essential for the detoxification of ROS and the reduction of oxidized proteins. Considering that the frequently mutated tumor suppressor and oncogenic pathways in cancers commonly lead to the increased accumulation of ROS (Szatrowski and Nathan, 1991; Lee et al., 1999; Vafa et al., 2002; Nogueira et al., 2008), the efficient detoxification of ROS is a requisite for cell proliferation.

Nrf2 directly activates the essential genes for the glutathione synthesis. The genes encoding the regulatory (GCLM) and catalytic (GCLC) subunits of γ-glutamylcysteinyl ligase, a rate-limiting enzyme for glutathione synthesis, are well known targets of Nrf2 (Moinova and Mulcahy, 1999; Solis et al., 2002; Bea et al., 2003; Sekhar et al., 2003). The gene encoding a subunit of the cystine transporter SLC7A11 (xCT) is another Nrf2 target (Sasaki et al., 2002), whose product increases the availability of cysteine for glutathione synthesis. Nrf2 also activates the genes encoding the four major NADPH producing enzymes, G6PD, PGD, ME1, and IDH1, for reducing oxidized glutathione and other cellular components. Thus, Nrf2 induces the production of glutathione and NADPH, conferring a growth advantage to cancer cells.

A recent study has demonstrated that an acute increase in the intracellular concentration of ROS inhibits the glycolytic enzyme pyruvate kinase M2 (PKM2) through the oxidation of Cys358 (Anastasiou et al., 2011). The inhibition of PKM2 redirects glucose into the pentose phosphate pathway and thereby generates NADPH for the detoxification of ROS. An increase in ROS levels also induces Nrf2-dependent transcription, thus the induction of the pentose phosphate pathway enzymes through Nrf2 could contribute to the redirection of glucose and NADPH production.

### NRF2 INHIBITORS FOR ANTICANCER THERAPY

Due to the multifaceted roles of Nrf2 in cancers, Nrf2 inhibitors could be effective for anticancer therapy. The suppression of antioxidant proteins, glutathione synthesis enzymes, and NADPH-producing enzymes would lead to ROS accumulation in cancer cells and subsequent oxidative damage to various intracellular components, thus compromising the cell viability. The simultaneous inhibition of the oxidative and non-oxidative arms of the pentose phosphate pathway is indeed effective for repressing tumor cell growth (Boros et al., 1997; Ramos-Montoya et al., 2006; Mitsuishi et al., 2012). Nrf2 inhibitors also sensitize cancers to the effects of chemotherapeutic drugs through the down-regulation of detoxification enzymes and drug excretion transporters (Singh et al., 2008; Wang et al., 2008b). While a number of Nrf2 inducers have been developed and tested in clinical trials (Pergola et al., 2011; Gold et al., 2012), few Nrf2 inhibitors have been developed. Brusatol has been purified from a plant extract of *Brucea javanica* (Simaroubaceae), which is an evergreen shrub grown in Southeast Asia and Northern Australia, and shown to inhibit ARE-luciferase activity and the protein accumulation of Nrf2 (Ren et al., 2011). However, the detailed mechanism by which brusatol enhances Nrf2 degradation and how it selectively inhibits the Keap1–Nrf2 pathway warrants further investigation.

Achieving specificity is the biggest challenge in the development of Nrf2 inhibitors. Nrf2 belongs to the CNC protein family, including NF-E2 p45, Nrf1, Nrf3, Bach1, and Bach2 (Motohashi et al., 2002). All the members form heterodimers with small Maf proteins through leucine zipper motifs and bind to the ARE consensus sequence through basic regions, suggesting that the bZip structure of Nrf2 shares many common properties with that of other CNC members. Thus, one of the targets for Nrf2 inhibitors would be the domain outside the bZip motif, and the other target would be the leucine zipper, as there are substantial variations in the leucine zipper compared with the basic regions in CNC family members.

In addition, achieving delivery specificity is also an important issue. The systemic inhibition of Nrf2 could exacerbate the side effects of chemo- and radiotherapies. Moreover, a recent study showed that Nrf2 deficiency in bone marrow cells aggravates metastasis (Satoh et al., 2010). Thus, a drug delivery method and protocol would need to be developed for the Nrf2 inhibitor to preferentially target cancer cells.

The positive feedback between Nrf2 and active PI3K–Akt signaling, which induces the malignant evolution of cancers, presents another area of interest for therapeutic development. An Nrf2 inhibitor would weaken the PI3K–Akt signaling activity in cancer cells, while inhibitors of PI3K–Akt signaling could antagonize Nrf2 activity. Thus, the disruption of the feedback activation represents an effective anti-cancer therapy. To decipher the functional interactions between Keap1–Nrf2 system and other oncogenic pathways is one of the most important assignments for the conquest of Nrf2-dependent cancers. 

## Conflict of Interest Statement

The authors declare that the research was conducted in the absence of any commercial or financial relationships that could be construed as a potential conflict of interest.
